# Integrating Non-Invasive Neurotherapies in Severe Depression: Where Does ECT Stand?

**DOI:** 10.1192/j.eurpsy.2026.11624

**Published:** 2026-07-31

**Authors:** C. E. Anghel, I. C. Băcilă

**Affiliations:** 1 Faculty of Medicine, The “Lucian Blaga” University; 2 ECT; 3 rTMS, Scientific Research Collective in Neurosciences - The “Dr. Gheorghe Preda” Clinical Hospital of Psychiatry, Sibiu, Romania

## Abstract

**Introduction:**

Major depressive disorder (MDD), particularly in its severe and treatment-resistant forms, remains a considerable clinical challenge despite advances in pharmacotherapy and psychotherapy. In recent years, non-invasive neurotherapies—such as repetitive transcranial magnetic stimulation (rTMS) and transcranial direct-current stimulation (tDCS)—have gained increasing attention due to their favorable safety profiles and expanding evidence. As these modalities become integrated into clinical pathways, the role of electroconvulsive therapy (ECT) requires renewed evaluation.

**Objectives:**

This presentation aims to critically compare ECT with emerging non-invasive neurotherapies and to outline an updated framework for their integration in the management of severe depression.

**Methods:**

This work draws on a narrative review of current clinical guidelines, recent research evidence, and implementation models related to ECT, rTMS, and tDCS published over the past five years. Clinical practice insights are incorporated, focusing on treatment sequencing, combination or stepwise approaches, and decision-making factors in real-world psychiatric settings.

**Results:**

ECT continues to demonstrate superior efficacy and speed of response in severe and treatment-resistant depression, while rTMS and tDCS offer greater tolerability and acceptance, with expanding indications. Differences are highlighted regarding cognitive effects, patient preference, long-term outcomes, and practical considerations for clinical deployment. Ethical and medico-legal aspects—including informed consent, patient autonomy, and shared decision-making—emerge as essential to fostering responsible, patient-centered use of neuromodulatory treatments.

**Conclusions:**

Integrating ECT within modern neurotherapeutic care requires a personalized, evidence-based approach that aligns clinical effectiveness with ethical practice. By combining current evidence, clinical insight, and evolving treatment models, a pragmatic framework is proposed to support clinicians in optimizing recovery, functioning, and quality of life for patients with severe depression. Further research and service development are needed to refine treatment pathways and enhance interdisciplinary neurotherapeutic care.

**Keywords:**

non-invasive neurotherapies, severe depression, ECT, personalized approach.

**Disclosure of Interest:**

None Declared

